# Association of Problematic Anger With Long-term Adjustment Following the Military-to-Civilian Transition

**DOI:** 10.1001/jamanetworkopen.2022.23236

**Published:** 2022-07-21

**Authors:** Amy B. Adler, Cynthia A. LeardMann, Javier Villalobos, Isabel G. Jacobson, David Forbes

**Affiliations:** 1 Center for Military Psychiatry and Neuroscience, Walter Reed Army Institute of Research, Silver Spring, Maryland; 2 Deployment Health Research Department, Naval Health Research Center, San Diego, California; 3 Leidos, Inc, San Diego, California; 4 Phoenix Australia-Centre for Posttraumatic Mental Health, Department of Psychiatry, University of Melbourne, Carlton, VIC, Australia

## Abstract

**Importance:**

Few studies have examined the role of problematic anger in long-term adjustment of service members transitioning out of the military.

**Objective:**

To determine the prevalence of problematic anger during the military-to-civilian transition period and the association of problematic anger with adjustment to civilian life.

**Design, Setting, and Participants:**

This cohort study used 2 waves of survey data administered approximately 5 years apart (time 1 [T1; September 26, 2014, to August 25, 2016] and time 2 [T2; October 23, 2019, to August 31, 2021]) from the Millennium Cohort Study, a population-based military study. Participants were US active-duty service members within 24 months of separating from military service at T1. Statistical analysis was performed from September 2021 to May 2022.

**Exposures:**

Problematic anger was operationalized as scoring at least 12 points on the 5-item Dimensions of Anger Reactions scale at T1.

**Main Outcomes and Measures:**

Behavioral and functional health (depression, posttraumatic stress disorder, problem drinking, functional limitations), relationship health (relationship quality, coping with parental demands, social support), and economic health (major financial problems, financial insecurity, homelessness, employment status) were assessed at T2. Covariates, assessed at T1, included demographics, military characteristics, mental health, problem drinking, and physical health.

**Results:**

Of the 3448 participants, 2625 (76.1%) were male, 217 (6.3%) were Hispanic, 293 (8.5%) were non-Hispanic Black, and 2690 (78.0%) were non-Hispanic White; the mean (SD) age was 40.1 (8.5) years; 826 (24.0%) met criteria for problematic anger. Prevalence of problematic anger was 15.9% (95% CI, 12.2%-19.7%) 24 months prior to military separation and 31.2% (95% CI, 26.2%-36.2%) 24 months following separation. After adjusting for covariates, problematic anger was associated with greater likelihood of behavioral and functional health outcomes (eg, posttraumatic stress disorder: adjusted odds ratio, 1.55, 95% CI, 1.23-1.96), relationship health difficulties (eg, low social support: aOR, 1.66; 95% CI, 1.23-2.24), and economic difficulties (eg, substantial financial insecurity: aOR, 1.64; 95% CI, 1.13-2.39) at T2.

**Conclusions and Relevance:**

This cohort study found an association between prevalence of problematic anger during the military-to-civilian transition and problematic anger with subsequent adjustment difficulties among US service members. These findings suggest the need to equip service members proactively with skills to identify and manage anger as a way to support them before and during this period of transition.

## Key Points

**Question:**

What is the association of problematic anger with long-term adjustment following the transition of US service members to civilian life?

**Findings:**

In this cohort study of 3448 active-duty service members transitioning out of the military, the prevalence of problematic anger 2 years after separation (31.2%) was nearly double the prevalence 2 years before separation (15.9%). Problematic anger was associated with behavioral health problems, relationship health concerns, and economic difficulties approximately 5 years later.

**Meaning:**

These findings suggest that problematic anger may be a determinant of maladjustment, offering a point of intervention for emotion regulation management to improve military-to-civilian transition.

## Teaser

This cohort study investigates the prevalence of problematic anger during the military-to-civilian transition period and the association of problematic anger with adjustment to civilian life among US military service members.

## Introduction

Although anger can be a useful emotion that signals the need to respond to a perceived injustice and promote social change, problematic anger is defined by increased distress and decreased functioning.^1^ Problematic anger has the potential to impede adjustment across different domains including behavioral health,^2,3^ social relationships,^1^ and economic functioning.^4^ More broadly, a recent study found problematic anger was associated with suicidal ideation and suicide attempts, after accounting for psychiatric risk factors.^5^ At present, however, problematic anger remains largely understudied, unrecognized, and undertreated.^6^

Substantial prevalence of problematic anger has been documented in military service members and veterans. In a large US study, 12% of currently serving personnel and 20% of veterans met criteria for problematic anger.^7^ Using the same measure, a large Australian study found 16% of actively serving personnel and 31% of recent military veterans met criteria for problematic anger. This near doubling of prevalence warrants attention given that separating from military service is fraught with risk.

Departing military service requires individuals to adapt their personal identity, social group, sense of purpose, economic situation, and employment.^8,9^ Studies have documented behavioral health risks associated with transitioning from military service^10,11,12^ as well as risks to adjustment associated with failure to participate in activities reflecting occupational and social engagement.^13^ Thus, prevalence of problematic anger during the transition from military service needs to be examined, taking a wide temporal lens given that transition is a process,^9^ and service members are encouraged to think about it 12 to 24 months in advance.

Furthermore, it is important to determine the degree to which problematic anger during the transition relates to adjustment difficulties in civilian life. At least one cross-sectional study demonstrated a link between problematic anger and economic distress (eg, financial strain, unemployment, homelessness), over and above demographics, military characteristics, and mental health.^4^ Given the study design, however, the degree to which problematic anger is a response or a contributor to difficult circumstances, or some combination of the two is unclear.^4^ Clarifying the temporal sequence of this association is critical to identifying periods of risk and opportunities for intervention. Such intervention can leverage advances in addressing problematic anger and potentially mitigate a cascade of ensuing detrimental outcomes.^14,15,16,17^

The present study analyzed 2 waves of data from a large cohort study to (1) characterize the prevalence of problematic anger in the 2 years preceding and following separation from military service and (2) identify the degree to which problematic anger is associated with adjustment approximately 5 years later across 3 domains (behavioral and functional health, relationship health, and economic health) while accounting for relevant covariates.

## Methods

### Population and Data Source

The Millennium Cohort Study is a population-based longitudinal study that investigates health effects associated with military service; the study’s methodology has been described in detail elsewhere.^18,19^ Briefly, service members representing all service branches and components (ie, active-duty, Reserves and National Guard personnel) were initially enrolled in 5 phases between July 1, 2001, and August 31, 2021. Cohort participants provide voluntary, informed consent, and they complete a survey at enrollment and are asked to complete a follow-up survey approximately every 3 to 5 years thereafter, even after they leave military service. The study protocol was approved by the Naval Health Research Center institutional review board. This study followed the Strengthening the Reporting of Observational Studies in Epidemiology (STROBE) reporting guideline.

A measure of problematic anger was introduced in the 2014 to 2016 survey. Therefore, the study population was restricted to participants (n = 74 232) who completed the 2014 to 2016 (time 1 [T1]) and 2019 to 2021 (time 2 [T2]) surveys (mean [SD] time between T1 and T2 was 5.0 years [0.51] years). The sample was further restricted to active-duty members who left military service within 2 years of the T1 survey (n = 3924). After excluding participants with missing data for separation (n = 20), missing 2 or more items on the Dimensions of Anger Reactions scale (DAR-5; n = 190), and missing covariate data (n = 266), 3448 participants were included in analyses (eFigure in Supplement 1).

### Timing and Type of Separation

**Figure 1.**
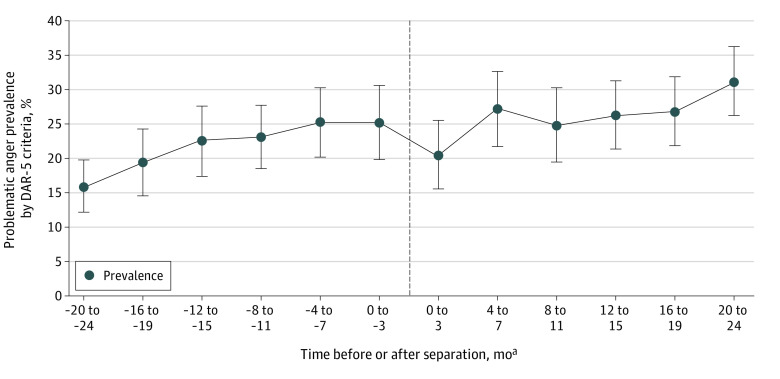
Prevalence of Problematic Anger by Subgroups Relative to Military Separation Date ^a^Zero month categorization determined by actual date of survey completion relative to separation date.

Timing of separation was determined using date of separation in relation to the date for T1 survey completion. Using 4-month intervals, participants were placed into 1 of 12 groups (ranging from 20 to 24 months preceding separation to 20 to 24 months following separation) (Figure 1).

Type of military separation was examined by grouping participants into categories based on separation codes received from Defense Manpower Data Center (DMDC). These codes indicated the primary reason for separating from military service and were grouped as follows: retirement, medical or disability, expiration of service, voluntary administrative leave, involuntary administrative leave, and disciplinary. In this sample, 25 (<1%) had an “other than honorable” discharge, thus we were not able to examine separation by type of discharge.

### Measures

Measures were assessed at T2 for the outcomes and T1 for covariates, unless otherwise specified. Outcome measure details are in eTable 1 in Supplement 1.

#### Behavioral and Functional Health

Depression was assessed with the 8-item Patient Health Questionnaire (PHQ-8) using Diagnostic and Statistical Manual of Mental Disorders (DSM) 4th edition^20^ criteria.^21^ Posttraumatic Stress Disorder (PTSD) was assessed by the PTSD Checklist-Civilian Version (PCL-C)^22^ at T1, and the PCL for DSM 5th edition (DSM-5; PCL-5) at T2. Responses from the PCL-C were recoded to align with corresponding PCL-5^23^ items, consistent with previous research.^24^

Problem drinking was assessed using alcohol-related PHQ items and defined as “yes” responses to at least one item.^25^ Functional limitations were assessed with 1 item estimating how many days participants were unable to work or perform their usual activities due to illness or injury, categorized into 5 groups.

#### Relationship Health

Relationship quality was assessed using 1 item, categorized as (1) unhappy or neutral and (2) happy.^26^ Coping with parental demands was assessed with 1 item about coping with day-to-day parental demands, categorized into 4 levels. Social support was assessed with 6 items from the Multidimensional Scale of Perceived Social Support^27^; scale means were categorized into low, medium, and high.^28^

#### Economic Difficulties

Major financial problems were assessed with 1 item.^4^ Financial security was assessed with 1 item asking about the “financial condition of you and your family,” categorized into 4 levels. Homelessness was assessed with 1 item asking if participants had slept in a shelter, on streets, or in other nonresidential settings because of having no other place to stay.^4,29^ Employment status was assessed with 1 item; responses included full-time, part-time, homemaker, retired, unemployed, looking for work, unemployed, not looking for work, unemployed, disabled, and student,^4^ which were collapsed into 6 groups.

#### Problematic Anger

Problematic anger was assessed with the 5-item DAR-5 (eg, “when I get angry, I get really mad”) at T1 (1 = not at all to 5 = very much), using the established cutoff of 12 points or higher. For those missing 1 item, mean imputation was used to calculate the sum score.^30^

#### Covariates

Demographic and military characteristics (sex, birthdate, race, ethnicity, military rank, service branch, deployment in support of operations in Iraq and Afghanistan) were obtained from the DMDC. Information on sex, race, and ethnicity was provided by service members using categories defined by the services. Combat experience was determined by 13 items (eg, “being attacked or ambushed,” “receiving small arms fire”) from T1 and previous Millennium Cohort surveys. For combat and deployment history, individuals were classified as nondeployed, deployed without combat, or deployed with combat experience.

Marital status, educational attainment, mental health, problem drinking, and physical health were assessed using T1 data. Consistent with the literature, physical health was categorized by percentiles based on the physical component summary (PCS) score from the Veterans Short Form-36 (SF-36V).^31,32,33^

### Statistical Analysis

Percentage screening positive for problematic anger was calculated for each of the timing and type of separation groups. Descriptive analyses compared problematic anger with each covariate and outcome. Nested logistic regression was performed to examine the associations between problematic anger and each outcome. Model 1 examined unadjusted associations; model 2 adjusted for demographics and military characteristics; model 3 further adjusted for behavioral and physical health factors (PTSD and/or depression, problem drinking, and PCS).

In supplemental analyses, model 4 further adjusted for timing and type of separation. Building on model 3, additional specific models adjusted for T1 social support (for the relationship health models) and T1 financial stress (for the economic difficulty models). To investigate the potential impact of the COVID-19 pandemic, an interaction between problematic anger and T2 survey date (ie, prepandemic [March 11, 2020, and earlier] or pandemic [after March 11, 2020]) was included in the fully adjusted models (model 3); models with a significant interaction term were stratified by T2 survey date. Because the PCL-5 includes 2 symptoms related to anger, we also conducted a sensitivity analysis removing these items from the T2 PTSD measure.

All analyses were conducted using SAS/STAT software version 9.4 (SAS Institute) from September 2021 to May 2022. Statistical significance was set at *P* < .05; all tests were 2-tailed.

## Results

Of the 3448 active-duty service members separating from the military, 2625 (76.1%) were male, 217 (6.3%) were Hispanic, 293 (8.5%) were non-Hispanic Black, 2690 (78.0%) were non-Hispanic White, and 2516 (73.0%) were married; the mean (SD) age was 40.1 (8.5) years. Descriptive and military characteristics of participants were summarized in Table 1.

**Table 1.** Characteristics by Problematic Anger Status at T1 Among Millennium Cohort Participants

Characteristic	Participants, No. (%)			
	Study sample (N = 3448)	No problematic anger (n = 2622)	Problematic anger (n = 826)
Age, mean (SD), y	40.1 (8.5)	41.0 (8.6)	37.1 (7.7)
Sex			
Male	2625 (76.1)	1999 (76.2)	626 (75.8)
Female	823 (23.9)	623 (23.8)	200 (24.2)
Race and ethnicity			
Hispanic or Latino	217 (6.3)	155 (5.9)	62 (7.5)
Non-Hispanic Black	293 (8.5)	212 (8.1)	81 (9.8)
Non-Hispanic White	2690 (78.0)	2073 (79.1)	617 (74.7)
Other^a^	248 (7.2)	182 (6.9)	66 (8.0)
Educational attainment			
High school or less	191 (5.5)	113 (4.3)	78 (9.4)
Some college or associate’s degree	1402 (40.7)	968 (36.9)	434 (52.5)
Bachelor’s degree	773 (22.4)	592 (22.6)	181 (21.9)
Graduate degree	1082 (31.4)	949 (36.2)	133 (16.1)
Marital status			
Single, never married	363 (10.5)	280 (10.7)	83 (10.1)
Married	2516 (73.0)	1957 (74.6)	559 (67.7)
Previously married	569 (16.5)	385 (14.7)	184 (22.3)
Military characteristics			
Military rank			
Enlisted	2145 (62.2)	1492 (56.9)	653 (79.1)
Officer	1303 (37.8)	1130 (43.1)	173 (20.9)
Service branch			
Army	1552 (45.0)	1064 (40.6)	488 (59.1)
Navy/Coast Guard	701 (20.3)	567 (21.6)	134 (16.2)
Marine Corps	293 (8.5)	204 (7.8)	89 (10.8)
Air Force	902 (26.2)	787 (30.0)	115 (13.9)
Combat and deployment^b^			
Not deployed	450 (13.1)	378 (14.4)	72 (8.7)
Deployed, no combat	556 (16.1)	486 (18.5)	70 (8.5)
Deployed with combat	2442 (70.8)	1758 (67.1)	684 (82.8)
Behavioral health			
Problem drinking^c^			
No	3150 (91.4)	2473 (94.3)	677 (82.0)
Yes	298 (8.6)	149 (5.7)	149 (18.0)
Mental health disorders^d^			
None	2806 (81.4)	2446 (93.3)	360 (43.6)
Depression	148 (4.3)	60 (2.3)	88 (10.7)
PTSD	190 (5.5)	64 (2.4)	126 (15.3)
Comorbid PTSD/depression	304 (8.8)	52 (2.0)	252 (30.5)
Physical health			
Physical component summary^e^			
Highest 15th percentile	530 (15.4)	449 (17.1)	81 (9.8)
Middle 70th percentile	2398 (69.6)	1867 (71.2)	531 (64.3)
Lowest 15th percentile	520 (15.1)	306 (11.7)	214 (25.9)

Abbreviations: PHQ, Patient Health Questionnaire; PTSD, posttraumatic stress disorder; T1, time 1 (September 26, 2014, to August 25, 2016).

^a^ Other includes individuals who identify as American Indian, Alaskan Native, or multiracial.

^b^ Assessed from electronic records obtained from the Defense Manpower Data Center combined with self-reported combat experience items on surveys up to the T1 survey.

^c^ Endorsement of 1 or more items on the PHQ alcohol items.

^d^ Assessed using the PHQ-8 and PTSD Checklist-Civilian Version items on the T1 survey.

^e^ Assessed using Physical Component Summary score of the Veterans Short Form-36, where higher scores indicate better health.

**Figure 2.**
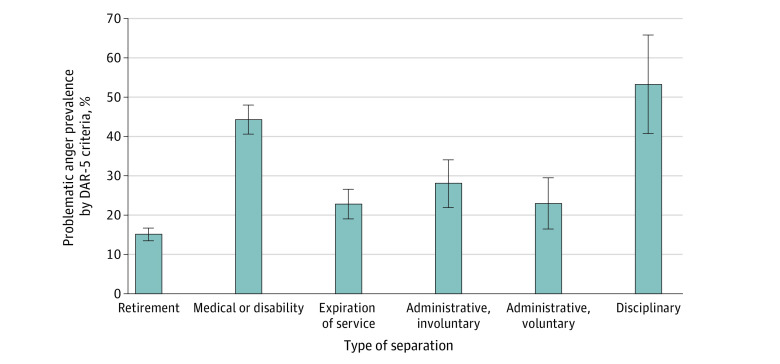
Prevalence of Problematic Anger by Type of Separation DAR-5 indicates Dimensions of Anger Reactions scale.

At T1, 826 (24.0%; 95% CI, 22.5%-25.4%) screened positive for problematic anger. Prevalence of problematic anger was lowest at 20 to 24 months prior to separation (15.9%; 95% CI, 12.2%-19.7%) and highest (31.2%; 95% CI, 26.2%-36.2%) at 20 to 24 months following separation, with a small dip in prevalence observed at 0 to 4 months immediately following separation (Figure 1). In terms of separation type, the highest prevalence was observed in the disciplinary (53.3%; 95% CI, 40.7%-66.0%) and medical or disability (44.4%; 95% CI, 40.7%-48.1%) groups, whereas problematic anger was lowest in the retirement (15.1%; 95% CI, 13.4%-16.7%) group (Figure 2).

Descriptive analyses indicated that participants with problematic anger were proportionally more likely to screen positive for each outcome in the 3 domains compared with those without problematic anger (Table 2). For example, those with problematic anger were proportionally more likely than those without problematic anger to have probable PTSD (412 of 825 participants [49.9%] vs 400 of 2619 participants [15.3%]), report poor or very poor coping with parental demands (74 of 545 participants [13.6%] vs 71 of 1448 participants [4.9%]), and report financial problems (114 of 821 participants [13.9%] vs 141 of 2615 participants [5.4%]).

**Table 2.** Outcomes at T2 by Problematic Anger Status at T1 Among Active-Duty Service Members

Outcome	No. (%)		
	Study sample	No problematic anger	Problematic anger
Behavioral and functional health			
Depression (n = 3444)^a^			
No	2907 (84.4)	2391 (91.3)	516 (62.6)
Yes	537 (15.6)	229 (8.7)	308 (37.4)
PTSD (n = 3444)^b^			
No	2632 (76.4)	2219 (84.7)	413 (50.1)
Yes	812 (23.6)	400 (15.3)	412 (49.9)
Problem drinking (n = 3446)^c^			
No	3075 (89.2)	2394 (91.3)	681 (82.7)
Yes	371 (10.8)	228 (8.7)	143 (17.4)
Days with functional limitations in last 12 mo (n = 3445)			
0-1	1013 (29.4)	844 (32.2)	169 (20.5)
2-5	781 (22.7)	632 (24.1)	149 (18.0)
6-15	754 (21.9)	576 (22.0)	178 (21.6)
16-60	498 (14.5)	331 (12.6)	167 (20.2)
>60	399 (11.6)	236 (9.0)	163 (19.7)
Relationship health			
Relationship quality (n = 2871)^d^			
Happy (5-7)	2337 (81.4)	1859 (84.2)	478 (72.1)
Unhappy/neutral (1-4)	534 (18.6)	349 (15.8)	185 (27.9)
Coping with parental demands (n = 1993)^e^			
Very well	674 (33.8)	577 (39.9)	97 (17.8)
Somewhat well	768 (38.5)	556 (38.4)	212 (38.9)
Fair	406 (20.4)	244 (16.9)	162 (29.7)
Poorly/very poorly	145 (7.3)	71 (4.9)	74 (13.6)
Social support (n = 3445)^f^			
High (mean 5-7)	2155 (62.6)	1788 (68.2)	367 (44.5)
Moderate (mean 3-5)	902 (26.2)	582 (22.2)	320 (38.8)
Low (mean 0-3)	388 (11.3)	251 (9.6)	137 (16.6)
Economic difficulties			
Major financial problems (n = 3436)			
No	3181 (92.6)	2474 (94.6)	707 (86.1)
Yes	255 (7.4)	141 (5.4)	114 (13.9)
Financial security (n = 3435)^g^			
Very comfortable/secure	1352 (39.4)	1161 (44.5)	191 (23.2)
Able to make ends meet	1342 (39.1)	1010 (38.7)	332 (40.3)
Occasional difficulty	486 (14.2)	296 (11.3)	190 (23.1)
Substantial financial insecurity	255 (7.4)	144 (5.5)	111 (13.5)
Homeless, last 6 y (n = 3424)			
No	3356 (98.0)	2581 (99.0)	775 (95.0)
Yes	68 (2.0)	27 (1.0)	41 (5.0)
Employment status (n = 3437)			
Full-time	2385 (69.4)	1902 (72.8)	483 (58.7)
Part-time	224 (6.5)	177 (6.8)	47 (5.7)
Not employed, looking	132 (3.8)	76 (2.9)	56 (6.8)
Not employed, not looking	206 (6.0)	157 (6.0)	49 (6.0)
Retired	285 (8.3)	203 (7.8)	82 (10.0)
Disabled	205 (6.0)	99 (3.8)	106 (12.9)

Abbreviations: PHQ, Patient Health Questionnaire; PTSD, posttraumatic stress disorder; T1, time 1 (September 26, 2014, to August 25, 2016); T2, time 2 (October 23, 2019, to August 31, 2021).

^a^ Assessed using the PHQ-8.

^b^ Assessed using the PTSD checklist for DSM-5 (PCL-5).

^c^ Endorsement of 1 or more items on the PHQ alcohol items.

^d^ Restricted to participants who reported being in a committed relationship at the time of survey completion.

^e^ Restricted to participants who reported having children at the time of survey completion.

^f^ Assessed using 6 items from the Multidimensional Scale of Perceived Social Support.

^g^ Substantial financial insecurity consisted of “tough to make ends meet” or “in over our heads.”

Unadjusted models revealed that problematic anger was significantly associated with each outcome (Table 3). Adjustment for demographics and military-related factors (model 2) attenuated the magnitudes of association; however, all associations remained statistically significant (Table 3).

**Table 3.** Associations of Behavioral and Functional Health, Relationship Health, and Economic Difficulties at T2 by Problematic Anger at T1 Among Service Members Who Separated From the Military

Outcome	Model 1: Unadjusted, OR (95% CI)	Model 2, aOR (95% CI)^a^	Model 3, aOR (95% CI)^b^
Behavioral and functional health			
Depression (n = 3444)^c^			
No	1 [Reference]	1 [Reference]	1 [Reference]
Yes	6.23 (5.12-7.58)	4.85 (3.95-5.96)	1.77 (1.37-2.30)
PTSD (n = 3444)^d^			
No	1 [Reference]	1 [Reference]	1 [Reference]
Yes	5.53 (4.65-6.58)	4.07 (3.39-4.88)	1.55 (1.23-1.96)
Problem drinking (n = 3446)^e^			
No	1 [Reference]	1 [Reference]	1 [Reference]
Yes	2.21 (1.76-2.76)	1.94 (1.53-2.47)	1.20 (0.88-1.63)
Days with functional limitations in last 12 mo (n = 3445)			
0-1	1 [Reference]	1 [Reference]	1 [Reference]
2-5	1.18 (0.92-1.50)	1.17 (0.91-1.51)	1.13 (0.85-1.50)
6-15	1.54 (1.22-1.95)	1.39 (1.09-1.78)	1.13 (0.85-1.50)
16-60	2.52 (1.97-3.23)	2.10 (1.62-2.73)	1.38 (1.02-1.88)
>60	3.45 (2.66-4.47)	2.65 (2.01-3.48)	1.13 (0.80-1.60)
Relationship health			
Relationship quality (n = 2871)^f^			
Happy (5-7)	1 [Reference]	1 [Reference]	1 [Reference]
Unhappy/neutral (1-4)	2.06 (1.68-2.53)	1.99 (1.60-2.47)	1.46 (1.12-1.90)
Coping with parental demands (n = 1993)^g^			
Very well	1 [Reference]	1 [Reference]	1 [Reference]
Somewhat well	2.27 (1.74-2.96)	2.19 (1.65-2.89)	1.96 (1.42-2.68)
Fair	3.95 (2.95-5.29)	3.19 (2.35-4.34)	2.36 (1.66-3.36)
Poorly/very poorly	6.20 (4.20-9.16)	4.71 (3.12-7.10)	2.64 (1.61-4.35)
Social support (n = 3445)^h^			
High (mean 5-7)	1 [Reference]	1 [Reference]	1 [Reference]
Moderate (mean 3-5)	2.68 (2.25-3.20)	2.36 (1.96-2.85)	1.69 (1.36-2.11)^i^
Low (mean 0-3)	2.66 (2.10-3.37)	2.36 (1.84-3.04)	1.66 (1.23-2.24)^i^
Economic difficulties			
Major financial problems (n = 3436)			
No	1 [Reference]	1 [Reference]	1 [Reference]
Yes	2.83 (2.18-3.67)	2.14 (1.62-2.82)	1.47 (1.05-2.06) ^j^
Financial security (n = 3435)^k^			
Very comfortable/secure	1 [Reference]	1 [Reference]	1 [Reference]
Able to make ends meet	2.00 (1.64-2.43)	1.50 (1.22-1.85)	1.30 (1.02-1.64)
Occasional difficulty	3.90 (3.08-4.95)	2.37 (1.83-3.07)	1.51 (1.11-2.05)
Substantial financial insecurity	4.69 (3.50-6.27)	2.92 (2.14-3.99)	1.64 (1.13-2.39)
Homeless, last 6 y (n = 3424)			
No	1.00	1.00	1.00
Yes	5.06 (3.09-8.28)	3.42 (2.03-5.77)	1.93 (0.99-3.76)
Employment status (n = 3437)			
Full-time	1 [Reference]	1 [Reference]	1 [Reference]
Part-time	1.05 (0.75-1.46)	0.97 (0.68-1.39)	0.83 (0.55-1.25)
Not employed, looking	2.90 (2.03-4.16)	2.52 (1.72-3.70)	1.61 (1.01-2.58)
Not employed, not looking	1.23 (0.88-1.72)	1.01 (0.71-1.44)	0.86 (0.57-1.31)
Retired	1.59 (1.21-2.10)	1.70 (1.27-2.29)	1.35 (0.95-1.92)
Disabled	4.22 (3.15-5.65)	2.98 (2.19-4.07)	1.12 (0.75-1.68)

Abbreviations: aOR, adjusted odds ratio; OR, odds ratio; PHQ, Patient Health Questionnaire; PTSD, posttraumatic stress disorder; T1, time 1 (September 26, 2014, to August 25, 2016); T2, time 2 (October 23, 2019, to August 31, 2021).

^a^ Adjusted for age, sex, race/ethnicity, educational attainment, marital status, military rank, service branch, and combat deployment history at T1.

^b^ Adjusted for variables in model 2, plus mental health (depression/PTSD), problem drinking, and physical health at T1.

^c^ Assessed using the PHQ-8.

^d^ Assessed using the PTSD checklist for DSM-5 (PCL-5).

^e^ Endorsement of 1 or more items on the PHQ alcohol items.

^f^ Restricted to participants who reported being in a committed relationship at the time of survey completion.

^g^ Restricted to participants who reported having children at the time of survey completion.

^h^ Assessed using 6 items from the Multidimensional Scale of Perceived Social Support.

^i^ When the pandemic interaction term (problematic anger × T2 survey date) was included in model 3, it was statistically significant (*P* = .03); once stratified by T2 survey date, the association between problematic anger and major social support was only significant among those completing the survey prepandemic (low social support, adjusted odds ratio: 1.87; 95% CI, 1.33-2.63; moderate social support: aOR, 1.98; 95% CI, 1.54-2.55).

^j^ When the pandemic interaction term (problematic anger × T2 survey date) was included in model 3, it was statistically significant (*P* = .03); once stratified by T2 survey date, the association between problematic anger and major financial problems was only significant among those completing the survey during the pandemic (aOR, 3.57; 95% CI, 1.64-7.76).

^k^ Substantial financial insecurity consisted of “tough to make ends meet” or “in over our heads.”

After further adjustment for mental health, problem drinking, and physical health at T1, 9 of 11 outcomes remained significant (Table 3). Problematic anger at T1 remained associated with elevated odds of probable depression (adjusted odds ratio [aOR], 1.77; 95% CI, 1.37-2.30) and probable PTSD (aOR, 1.55; 95% CI, 1.23-1.96); when the 2 anger items were removed from the PCL-5, the PTSD results remained statistically significant (aOR, 1.57; 95% CI, 1.25-1.99). For functional limitations, 1 category remained significant (16 to 60 days: aOR, 1.38; 95% CI, 1.02-1.88). For relationship health, relationship quality (aOR, 1.46; 95% CI, 1.12-1.90), parental coping (“somewhat well”: aOR, 1.96; 95% CI, 1.42-2.68; “fair”: aOR, 2.36; 95% CI, 1.66-3.36; “poorly” or “very poorly”: aOR, 2.64; 95% CI, 1.61-4.35), and social support (“moderate”: aOR, 1.69; 95% CI, 1.36-2.11; “low”: aOR, 1.66; 95% CI, 1.23-2.24) remained significant. After further adjustment for T1 social support, there was modest attenuation, but results remained generally consistent (eTable 2 in Supplement 1). For the final model 3 outcomes, major financial problems (aOR, 1.47; 95% CI, 1.05-2.06), lower financial security (“able to make ends meet”: aOR, 1.30; 95% CI, 1.02-1.64; “occasional difficulty”: aOR, 1.51; 95% CI, 1.11-2.05; substantial financial insecurity: aOR, 1.64; 95% CI, 1.13-2.39), and being unemployed, looking for work (aOR, 1.61; 95% CI, 1.01-2.58) remained significant. After further adjustment for T1 financial stress, the magnitude of the associations was attenuated, but relatively consistent (eTable 2 in Supplement 1). In supplemental analyses, aORs and statistical significance for behavioral, functional, and relationship health outcomes were largely similar when adjusted for timing and type of separation (model 4; eTable 3 in Supplement 1); however, the associations between problematic anger and economic difficulties were attenuated and no longer significant.

Within the context of the pandemic, 2556 (74.1%) completed the survey prepandemic and 892 (25.9%) during the pandemic. When the pandemic interaction term was included in the model 3 analyses (data not shown), 2 of the 11 interaction terms were statistically significant. The association of problematic anger with major financial problems was only significant among those completing the survey during the pandemic (aOR, 3.17; 95% CI, 1.45-6.92), whereas social support was only significant among those completing the survey prepandemic (low social support: aOR: 1.87; 95% CI, 1.33-2.63; moderate social support: aOR, 1.98; 95% CI, 1.54-2.55).

## Discussion

In this cohort study, prevalence of problematic anger generally increased in the months leading up to and after separation from military service. Problematic anger during this transition period was associated with difficulties in behavioral, functional, relationship, and economic health 5 years later, even after adjusting for covariates, including behavioral health. These findings suggest that problematic anger needs to be considered in understanding military-to-civilian adjustment. To our knowledge, this is the first study to characterize problematic anger during the transition from military service and to document its association with subsequent community reintegration.

Consistent with findings from an Australian study,^34^ prevalence of problematic anger was approximately twice as high in the subgroup assessed at 20 to 24 months following separation compared with the subgroup assessed at 20 to 24 months preseparation. The rise in prevalence is most apparent early and late in the transition phase, whereas prevalence within the 1-year window surrounding the date of separation appears relatively stable. This pattern highlights the importance of considering the military-to-civilian transition from a relatively wide temporal lens. Interestingly, the dip in prevalence at the point of separation suggests there may be a honeymoon period of optimism or relief, but this reprieve appears temporary as prevalence rebounds 4 months later.

Programs designed to promote successful adaptation during the military-to-civilian transition should target preseparation and the immediate postseparation period; however, these programs should also continue to provide support even years later, given there was no observed levelling off of prevalence 2 years after separation. Unsurprisingly, problematic anger at transition was most prevalent among service members separating for disciplinary and medical or disability reasons. These results can inform how resources are targeted for groups particularly at risk.

Beyond these descriptive results, findings from adjusted models suggest that problematic anger is associated with subsequent behavioral and functional health, such as depression and PTSD. These findings were evident even after adjusting for T1 behavioral health, and timing and type of separation. Interestingly, this pattern did not fully extend to problem drinking, suggesting that T1 behavioral health difficulties may be more useful than problematic anger in understanding subsequent hazardous alcohol use. Although not examined here, problematic anger may also be associated with suicide-related outcomes given that previous findings demonstrate a link between problematic anger and suicidal ideation and attempts.^5^

Findings regarding problematic anger’s association with unhealthy relationships are particularly noteworthy. Problematic anger appears to impede relationships with spouses, partners, and children even after accounting for behavioral health and timing and type of separation. To our knowledge, these are among the first analyses to demonstrate the long-term toll of problematic anger on meaningful relationships, social connection, and parenting. These associations highlight the potential intergenerational impact of problematic anger.

Finally, the present study extends cross-sectional data linking problematic anger with economic distress.^4^ Problematic anger appears to place individuals at risk for subsequent financial uncertainty and unemployment, although these associations may be accounted for in part by T1 financial stress and timing and type of separation. Results also demonstrated a link with homelessness, although these findings did not reach significance after adjusting for T1 behavioral health. Future research should expand the assessment of homelessness to include unstable housing and doubling up in housing to determine if an independent relationship between problematic anger and homelessness emerges.

We addressed the generalizability of findings to the pandemic given that T2 surveys were administered both before and during the COVID-19 pandemic, which was accompanied by heightened anxiety^35,36^ and economic distress.^37^ Overall, the associations between problematic anger and outcomes did not appear to differ by pandemic period, suggesting the pandemic had a minimal influence on these relationships. However, problematic anger was only associated with social support prepandemic and economic stress during the pandemic. During periods of extreme societal stress, social support may be impacted for everyone, but problematic anger may introduce additional financial vulnerability.

### Limitations

Although the present study relied on 2 waves of data separated by several years, the data are observational and cannot demonstrate causation. Some subgroups were small, potentially suppressing identification of significant associations. The economic outcome measures were based on 1-item subjective assessments and may not fully capture the desired constructs. In addition, the T2 homelessness time frame (6 years) could have potentially overlapped with the T1 survey, although a post hoc sensitivity analysis restricting the sample to those who had not reported homelessness at T1 demonstrated results were essentially unchanged (aOR, 1.92; 95% CI, 0.91-4.02). Analyses examining potential differences by pandemic period may be confounded by demographic and other factors associated with participants who are late survey responders.^38^

## Conclusions

The fact that problematic anger is associated with a range of difficulties in adjustment years later even after accounting for T1 behavioral health indicates that problematic anger may serve as a critical warning sign for individuals and organizations. Individuals may benefit from knowing that problematic anger may pose a risk to their well-being, important relationships, and fiscal health. Much like public health messaging regarding diet and exercise can encourage individuals to follow a healthier lifestyle,^39,40^ warning about the association of problematic anger on well-being may be similarly helpful; military-to-civilian transition programs could proactively address problematic anger and its possible cost to veterans. Given problematic anger is often underrecognized and undertreated,^6^ organizations could also leverage these results by ensuring that service members are trained in healthy emotion regulation, and first-line leaders are trained to encourage emotion regulation in teams. Training could also target individuals at high risk for problematic anger, and screening for problematic anger could be considered prior to military accession. Future research should examine the association between problematic anger and suicide risk in the military-to-civilian transition context, the development of problematic anger, and how prevention and intervention programs can disrupt the emergence of problematic anger in the military and other high-risk occupations where anger may be perceived as integral to the occupational culture.^41^

## Notes

Supplement 1. **eFigure.** Study Flow Diagram **eTable 1.** Description of Measures Assessing Health Outcomes **eTable 2.** Models Adjusting for Social Support and Financial Difficulties **eTable 3.** Models Adjusting for Timing and Type of Separation

Supplement 2.
Nonauthor Collaborators
