# Supplementary material for: Association of Problematic Anger With Long-term Adjustment Following the Military-to-Civilian Transition
Source: JAMA Netw Open. 2022 Jul 21;5(7):e2223236. doi: 10.1001/jamanetworkopen.2022.23236 (PMC9305378; doi:10.1001/jamanetworkopen.2022.23236)
Supplement: Supplement 1. — eFigure. Study Flow Diagram eTable 1. Description of Measures Assessing Health Outcomes eTable 2. Models Adjusting for Social Support and Financial Difficulties eTable 3. Models Adjusting for Timing and Type of Separation [file jamanetwopen-e2223236-s001.pdf]

Supplemental Online Content

Adler AB, LeardMann CA, Villalobos J, Jacobson IG, Forbes D, for the Millennium Cohort Study Team. Association of problematic anger with long-term adjustment following the military-to-civilian transition. *JAMA Netw Open.* 2022;5(7):e2223236. doi:10.1001/jamanetworkopen.2022.23236

eFigure. Study Flow Diagram

eTable 1. Description of Measures Assessing Health Outcomes

eTable 2. Models Adjusting for Social Support and Financial Difficulties

eTable 3. Models Adjusting for Timing and Type of Separation

This supplemental material has been provided by the authors to give readers additional information about their work.

eFigure. Study Flow Diagram

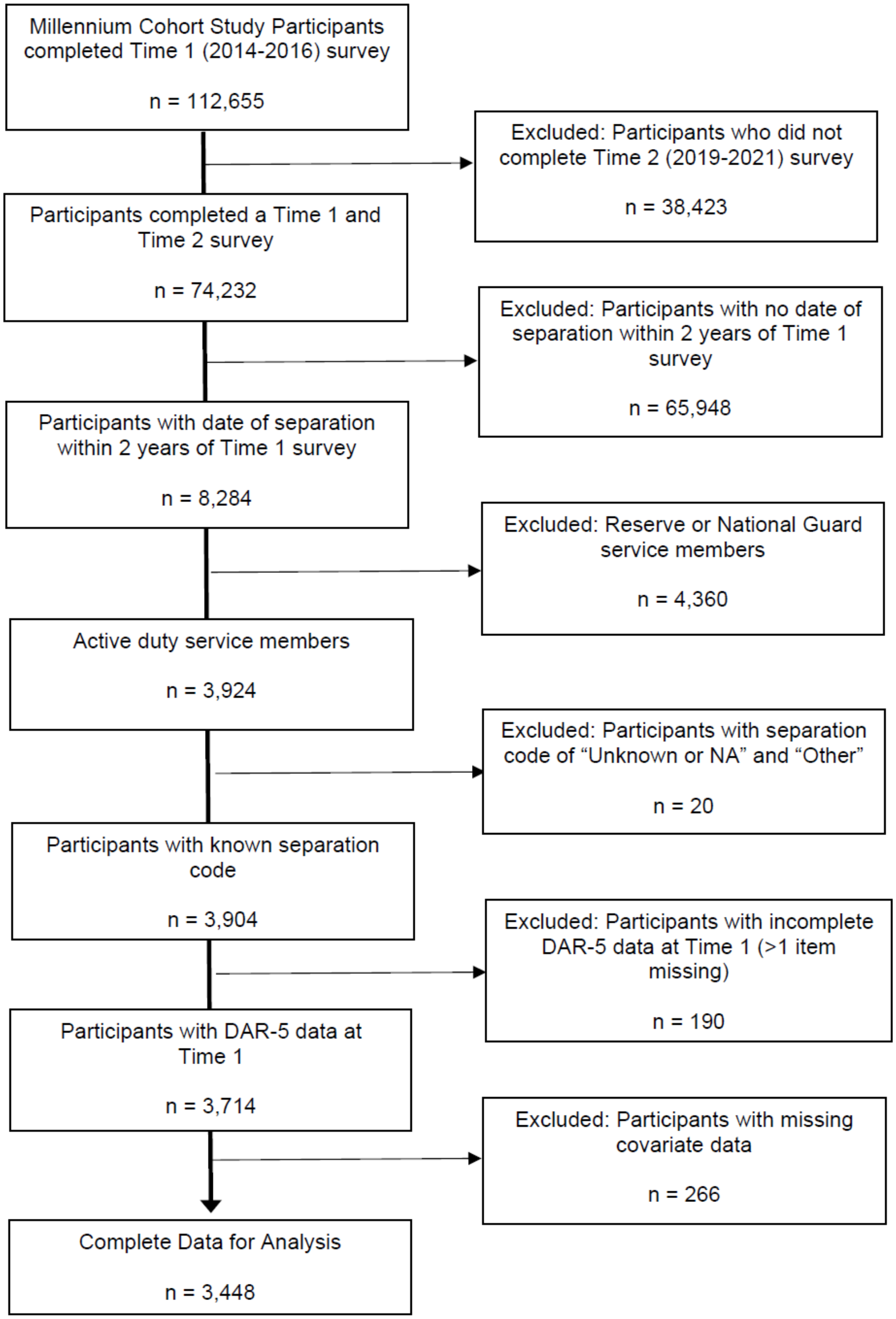

© 2022 Adler AB et al. *JAMA Network Open.*

eTable 1. Description of Measures Assessing Health Outcomes

Outcome	Description of Items	Response Options	Scoring
**Behavioral and Functional Health**			
Depression (Patient Health Questionnaire depression scale, PHQ-8)	8 items, measuring frequency of depression symptoms in the last 2 weeks.<br>Sample items:<br>• *Little interest or pleasure in doing things*<br>• *Feeling down, depressed, or hopeless*	• Not at all<br>• Several days<br>• More than half the days<br>• Nearly every day	Positive screen for major depressive disorder based on DSM-IV criteria (ie, endorsement of 5 of the 8 PHQ items for "more than half the days" or more frequently, including anhedonia and depressed mood) (no/yes) (per standard scoring)
Posttraumatic stress disorder (PTSD Checklist–DSM-5, PCL-5)	20 items, measuring the frequency of PTSD symptoms in the past month.<br>Sample items:<br>• *Repeated, disturbing, and unwanted memories of the stressful experience*<br>• *Feeling distant or cut off from other people*	• Not at all<br>• A little bit<br>• Moderately<br>• Quite a bit<br>• Extremely	Positive screen for PTSD based on DSM-5 criteria (i.e., endorsement of at least 1 intrusion, 1 avoidance, 2 negative alterations in cognitions or mood, and 2 hyperarousal symptoms) (no/yes) (per standard scoring)
Problem drinking (PHQ alcohol scale)	5 items, assessing problematic alcohol-related behaviors in past 12 months.<br>Sample items:<br>• *You drank alcohol even though a doctor suggested that you stop drinking because of a problem with your health.*<br>• *You had a problem getting along with people while you were drinking*	• No<br>• Yes	Positive screen for problem drinking behaviors based on endorsement of at least 1 item (no/yes) (per standard scoring)
Functional limitations	1 item ("Over the past 12 months, approximately how many days were you unable to work or perform your usual activities because of illness or injury? Exclude lost time for pregnancy and childbirth.")	• None<br>• 1 days<br>• 2 to 5 days<br>• 6 to 10 days<br>• 11 to 15 days<br>• 16 to 20 days<br>• 21 to 60 days<br>• > 60 days	To balance groups, responses categorized as:<br>• 0-1 days<br>• 2-5 days<br>• 6-15 days<br>• 16-60 days<br>• > 60 days
**Relationship Health**			
Relationship Quality (National Survey of Family and Households)	1 item ("Taking things all together, how would you describe your relationship with your significant other?")	1 ("very unhappy") to 7 ("very happy"), options 2 through 6 not labelled	Categorized as:<br>• unhappy/neutral (1-4)<br>• happy (5-7)

© 2022 Adler AB et al. *JAMA Network Open.*

			Categories collapsed due to low frequency of endorsement for some response options
Coping with parental demands	1 item ("In general, how well do you feel you are coping with the day-to-day demands of parenthood/raising children?")	• Very poorly<br>• Poorly<br>• Fair<br>• Somewhat well<br>• Very well	Categorized as:<br>• Poorly/very poorly<br>• Fair<br>• Somewhat well<br>• Very well<br>Categories collapsed due to low frequency of endorsement for some response options
Social support (Multidimensional Scale of Perceived Social Support)	6 items, assessing social support<br>Sample items:<br>• *I get the emotional help and support I need from my family.*<br>• *I can talk about my problems with my friends.*	• Very strongly disagree (1)<br>• Strongly disagree (2)<br>• Mildly disagree (3)<br>• Neutral (4)<br>• Mildly agree (5)<br>• Strongly agree (6)<br>• Very strongly agree (7)	Mean score calculated, categorized as:<br>• Low (0 to 3)<br>• Medium (3 to 5)<br>• High (5 to 7)<br>Per standard scoring
**Economic Difficulties**			
Major financial problems	1 item, assessed as "suffered major financial problems (such as bankruptcy)" over the last 3 years	• No<br>• Yes	Categorized as no/yes
Financial security	1 item ("Which best describes the financial condition of you and your family?")	• Very comfortable and secure<br>• Able to make ends meet without much difficulty<br>• Occasionally have some difficulty making ends meet<br>• Tough to make ends meet but keeping our heads above water<br>• In over our heads	Categorized as:<br>• Very comfortable and secure<br>• Able to make ends meet<br>• Occasional difficulty<br>• Substantial financial insecurity (tough to make ends meet and in over our heads)<br><br>Categories collapsed due to low frequency of endorsement for some response options
Homeless, last 6 years	1 item ("At any time in the last 6 years have you found it necessary to sleep in a shelter, on the streets, or in another non-residential setting because of having no other place to stay? Please only refer to instances during or after military service.")	• No<br>• Yes	Categorized as no/yes

© 2022 Adler AB et al. *JAMA Network Open.*

Employment status	1 item ("Which of the following best describes your employment status?")	• Full-time (≥30 hours/week)<br>• Part-time (<30 hours/week)<br>• Not employed, looking<br>• Not employed, not looking<br>• Not employed, retired<br>• Not employed, disabled<br>• Homemaker<br>• Other	Categorized as:<br>• Full-time<br>• Part-time<br>• Not employed, looking<br>• Not employed, not looking<br>• Retired<br>• Disabled<br>• Retired<br>Categories collapsed due to low frequency of endorsement for some response options

© 2022 Adler AB et al. *JAMA Network Open.*

eTable 2. Models Adjusting for Social Support and Financial Difficulties			
	Model 3: adjusted for demographics, military characteristics, behavioral health, and physical health at T1[a]	Model 3 + adjusted for social support at T1[b]	Model 3 + adjusted for financial stress at T1[c]
	AOR (95% CI)	AOR (95% CI)	AOR (95% CI)
**Relationship Health**			
Relationship quality[d] (n=2,871)			
Happy (5-7)	1.00	1.00	
Unhappy/neutral (1-4)	1.46 (1.12, 1.90)	1.17 (0.89, 1.54)	
Coping with parental demands[e] (n=1,993)			
Very well	1.00	1.00	
Somewhat well	1.96 (1.42, 2.68)	1.78 (1.28, 2.45)	
Fair	2.36 (1.66, 3.36)	2.02 (1.41, 2.91)	
Poorly/very poorly	2.64 (1.61, 4.35)	1.93 (1.15, 3.23)	
Social support[f] (n=3,445)			
High (mean 5-7)	1.00	1.00	
Moderate (mean 3-5)	1.69 (1.36, 2.11)[g]	1.28 (1.01, 1.61)	
Low (mean 0-3)	1.66 (1.23, 2.24)[g]	1.18 (0.86, 1.61)	
**Economic Difficulties**			
Major financial problems (n=3,436)			
No	1.00		1.00
Yes	1.47 (1.05, 2.06)[h]		1.41 (1.00, 1.98)
Financial security[i] (n=3,435)			
Very comfortable/secure	1.00		1.00
Able to make ends meet	1.30 (1.02, 1.64)		1.30 (1.02, 1.65)
Occasional difficulty	1.51 (1.11, 2.05)		1.46 (1.07, 1.99)
Substantial financial insecurity	1.64 (1.13, 2.39)		1.59 (1.09, 2.33)
Homeless, last 6 years (n=3,424)			
No	1.00		1.00
Yes	1.93 (0.99, 3.76)		1.81 (0.93, 3.55)
Employment (n=3,437)			
Full-time	1.00		1.00
Part-time	0.83 (0.55, 1.25)		0.83 (0.55, 1.25)
Not employed, looking	1.61 (1.01, 2.58)		1.57 (0.98, 2.52)
Not employed, not looking	0.86 (0.57, 1.31)		0.86 (0.56, 1.31)
Retired	1.35 (0.95, 1.92)		1.34 (0.94, 1.90)
Disabled	1.12 (0.75, 1.68)		1.10 (0.73, 1.65)

[a] Adjusted for age, sex, race/ethnicity, educational attainment, marital status, military rank, service branch, and combat deployment history, mental health (depression/PTSD), problem drinking, and physical health. AOR and 95% CI are identical to those Table 3. Displayed to support interpretation of the results.
[b] Adjusted for variables in Model 3 plus T1 social support.
[c] Adjusted for variables in Model 3 plus T1 financial status.
[d] Restricted to participants who reported being in a committed relationship at the time of survey completion.
[e] Restricted to participants who reported having children at the time of survey completion.
[f] Assessed using 6 items from the Multidimensional Scale of Perceived Social Support.
[g] When the pandemic interaction term (problematic anger*T2 survey date) was included in Model 3, it was statistically significant (p=.03); once stratified by T2 survey date, the association between problematic anger and major social support was only significant among those completing the survey pre-pandemic (low social support, AOR: 1.87, 95% CI: 1.33, 2.63; moderate social support, AOR: 1.98, 95% CI: 1.54, 2.55).
[h] When the pandemic interaction term (problematic anger*T2 survey date) was included in Model 3, it was statistically significant (p=.03); once stratified by T2 survey date, the association between problematic anger and major financial problems was only significant among those completing the survey during the pandemic (AOR: 3.57, 95% CI: 1.64, 7.76).
[i] Substantial financial insecurity consisted of “tough to make ends meet” or “in over our heads”.

eTable 3. Models Adjusting for Timing and Type of Separation		
	Model 3: adjusted for demographics, military characteristics, behavioral health, and physical health at T1[a]	Model 4: Model 3 + adjusted for timing and type of separation[b]
	AOR (95% CI)	AOR (95% CI)
**Behavioral and Functional Health**		
Depression[c] (n=3,444)		
No	1.00	1.00
Yes	1.77 (1.37, 2.30)	1.70 (1.31, 2.22)
PTSD[d] (n=3,444)		
No	1.00	1.00
Yes	1.55 (1.23, 1.96)	1.50 (1.19, 1.90)
Problem drinking[e] (n=3,446)		
No	1.00	1.00
Yes	1.20 (0.88, 1.63)	1.17 (0.85, 1.60)
Functional limitations, last 12 months (n=3,445)		
0-1 days	1.00	1.00
2-5 days	1.13 (0.85, 1.50)	1.12 (0.84, 1.49)
6-15 days	1.13 (0.85, 1.50)	1.09 (0.82, 1.45)
16-60 days	1.38 (1.02, 1.88)	1.32 (0.97, 1.80)
>60 days	1.13 (0.80, 1.60)	1.05 (0.74, 1.49)
**Relationship Health**		
Relationship quality[f](n=2,871)		
Happy (5-7)	1.00	1.00
Unhappy/neutral (1-4)	1.46 (1.12, 1.90)	1.46 (1.12, 1.90)
Coping with parental demands[g] (n=1,993)		
Very well	1.00	1.00
Somewhat well	1.96 (1.42, 2.68)	1.95 (1.41, 2.68)
Fair	2.36 (1.66, 3.36)	2.34 (1.64, 3.35)
Poorly/very poorly	2.64 (1.61, 4.35)	2.72 (1.64, 4.51)
Social support[h] (n=3,445)		
High (mean 5-7)	1.00	1.00
Moderate (mean 3-5)	1.69 (1.36, 2.11)[i]	1.70 (1.36, 2.12)
Low (mean 0-3)	1.66 (1.23, 2.24)[i]	1.66 (1.23, 2.24)
**Economic Difficulties**		
Major financial problems (n=3,436)		
No	1.00	1.00
Yes	1.47 (1.05, 2.06)[j]	1.35 (0.96, 1.89)
Financial security[k] (n=3,435)		
Very comfortable/secure	1.00	1.00
Able to make ends meet	1.30 (1.02, 1.64)	1.25 (0.98, 1.59)
Occasional difficulty	1.51 (1.11, 2.05)	1.36 (0.99, 1.86)
Substantial financial insecurity	1.64 (1.13, 2.39)	1.46 (1.00, 2.14)
Homeless, last 6 years (n=3,424)		
No	1.00	1.00
Yes	1.93 (0.99, 3.76)	1.77 (0.90, 3.49)
Employment status[l] (n=3,437)		
Full-time	1.00	1.00
Part-time	0.83 (0.55, 1.25)	0.83 (0.55, 1.26)
Not employed, looking	1.61 (1.01, 2.58)	1.59 (0.99, 2.55)
Not employed, not looking	0.86 (0.57, 1.31)	0.82 (0.53, 1.25)
Retired	1.35 (0.95, 1.92)	1.59 (1.10, 2.31)

© 2022 Adler AB et al. *JAMA Network Open.*

Disabled	1.12 (0.75, 1.68)	1.00 (0.66, 1.52)

Abbreviations: PTSD, posttraumatic stress disorder; PHQ, Patient Health Questionnaire
[a] Adjusted for age, sex, race/ethnicity, educational attainment, marital status, military rank, service branch, and combat deployment history, mental health (depression/PTSD), problem drinking, and physical health. AOR and 95% CI are identical to those in Table 3. Displayed to support interpretation of the results.
[b] Adjusted for variables in Model 3 plus timing of separation (4-month intervals) and type of separation.
[c] Assessed using the eight PHQ depression items.
[d] Assessed using the PTSD checklist for DSM-5 (PCL-5).
[e] Endorsement of one or more item on the PHQ alcohol items.
[f] Restricted to participants who reported being in a committed relationship at the time of survey completion.
[g] Restricted to participants who reported having children at the time of survey completion.
[h] Assessed using 6 items from the Multidimensional Scale of Perceived Social Support.
[i] When the pandemic interaction term (problematic anger*T2 survey date) was included in Model 3, it was statistically significant (p=.03); once stratified by T2 survey date, the association between problematic anger and major social support was only significant among those completing the survey pre-pandemic (low social support, AOR: 1.87, 95% CI: 1.33, 2.63; moderate social support, AOR: 1.98, 95% CI: 1.54, 2.55).
[j] When the pandemic interaction term (problematic anger*T2 survey date) was included in Model 3, it was statistically significant (p=.03); once stratified by T2 survey date, the association between problematic anger and major financial problems was only significant among those completing the survey during the pandemic (AOR: 3.57, 95% CI: 1.64, 7.76).
[k] Substantial financial insecurity consisted of “tough to make ends meet” or “in over our heads”.
[l] Type of separation categories were collapsed (disciplinary with administrative involuntary; administrative voluntary with expiration of service) due to small cell sizes, so that the model would converge.

© 2022 Adler AB et al. *JAMA Network Open.*
