# Supplementary material for: Association of Problematic Anger With Long-term Adjustment Following the Military-to-Civilian Transition
Source: JAMA Netw Open. 2022 Jul 21;5(7):e2223236. doi: 10.1001/jamanetworkopen.2022.23236 (PMC9305378; doi:10.1001/jamanetworkopen.2022.23236)
Supplement: Supplement 2. — Nonauthor Collaborators [file jamanetwopen-e2223236-s002.pdf]

Supplemental Online Content: Nonauthor Collaborators
*First name, last name, and suffix (if applicable) are required and will appear in PubMed.

*Group Name(s): Millennium Cohort Study Team							
***First Name and Middle Initial(s)**	***Last Name**	***Suffix (eg, Jr, III)**	Academic Degrees	Institution	Location (city, state/province, country)	Role or Contribution, eg, chair, principal investigator	Group (if more than 1 Group listed in the byline) and/or Subgroup (eg, Steering Committee)
Jenn	Belding		PhD	Naval Health Research Center	San Diego, CA, USA	Associate Investigator	
Satbir	Boparai		MBA	Naval Health Research Center	San Diego, CA, USA	Data Manager	
Felicia	Carey		PhD	Naval Health Research Center	San Diego, CA, USA	Associate Investigator	
Sheila	Castañeda		PhD	Naval Health Research Center	San Diego, CA, USA	Associate Investigator	
Toni Rose	Geronimo-Hara		MPH	Naval Health Research Center	San Diego, CA, USA	Data Analyst	
Claire	Kolaja		MPH	Naval Health Research Center	San Diego, CA, USA	Associate Investigator	
Sandra	Magallon		MPH	Naval Health Research Center	San Diego, CA, USA	Research Assistant	
Anna	Rivera		MPH	Naval Health Research Center	San Diego, CA, USA	Associate Investigator	
Rudolph	Rull		PhD	Naval Health Research Center	San Diego, CA, USA	Principal Investigator	
Julia	Seay		BS	Naval Health Research Center	San Diego, CA, USA	Associate Investigator	
Neika	Sharifian		PhD	Naval Health Research Center	San Diego, CA, USA	Associate Investigator	
Beverly	Sheppard		BS	Naval Health Research Center	San Diego, CA, USA	Study Coordinator	
Daniel	Trone		PhD	Naval Health Research Center	San Diego, CA, USA	Associate Investigator	
Jennifer	Walstrom			Naval Health Research Center	San Diego, CA, USA	Study Coordinator	
Nikki	Wooten		PhD	Naval Health Research Center	San Diego, CA, USA	Associate Investigator	
Katie	Zhu		MPH	Naval Health Research Center	San Diego, CA, USA	Data Analyst	
